# Serum concentrations and pharmacokinetics of linezolid in critically ill patients dialyzed by ADVanced Organ Support compared to conventional continuous renal replacement therapy

**DOI:** 10.1007/s00228-026-04129-0

**Published:** 2026-07-10

**Authors:** Julian Triebelhorn, Johanna Erber, Heike Schneider, Jochen Schneider, Laura Wagner, Yizhu Li, Eva Ortner, Roland M. Schmid, Ulrich Mayr, Tobias Lahmer, Miriam Dibos

**Affiliations:** 1 TUM School of Medicine and Health- Clinical Department of Internal Medicine II, TUM University Hospital, Ismaninger Str. 22, Munich, 81675 Germany; 2 TUM School of Medicine and Health- Clinical Department of Clinical Chemistry and Laboratory Medicine, TUM University Hospital, Munich, Germany

**Keywords:** Linezolid, Dialysis, Clearance, ADVOS, ICU

## Abstract

**Background:**

Dosing of antibiotics proves to be both crucial and especially challenging in critically ill patients due to extracorporeal therapies. In this exploratory study, we compared the influence of the albumin based ADVanced Organ Support (ADVOS) system to conventional continuous venovenous hemodialysis (CVVHD) on linezolid serum concentrations.

**Methods:**

A total of 14 critically ill patients (8 ADVOS, 6 CVVHD) receiving linezolid 600 mg twice daily were analyzed, comprising 22 measurement cycles. Machine clearance was calculated by pre- and post-filter concentrations; total and patient clearance were estimated by area under the curve (AUC) calculations. Linezolid pharmacokinetics were evaluated by C_min_ and estimated AUC_24_, considering target ranges. All parameters were compared between dialysis types.

**Results:**

Machine clearance was significantly higher in ADVOS than in CVVHD (median 3.4 vs. 1.6 L/h, *p* < 0.001), accounting for a higher share of total clearance (38.1% vs. 18.2%). Total clearance (9.3 vs. 10.4 L/h) and patient clearance (6.7 vs. 8.4 L/h) did not differ significantly, nor did AUC24 (250.9 vs. 221.2 mg·h/L) or Cmin (4.6 vs. 4.0 mg/L). A substantial proportion of subtherapeutic serum concentrations was observed in both groups (45.5% by AUC24 and 31.8% by Cmin).

**Conclusion:**

This in-vivo study provides real-life data of linezolid levels in ICU-patients treated with conventional CVVHD and ADVOS. The high occurrence of subtherapeutic serum concentrations and a significant difference in dialysis capacities depending on dialysis type indicate the necessity of a tailored therapeutic drug monitoring approach in this vulnerable population.

**Supplementary Information:**

The online version contains supplementary material available at 10.1007/s00228-026-04129-0.

## Background

Infections are one of the leading causes of death in critically ill patients [1]. Dosing of adequate antibiotic treatment is often challenged by altered pharmacokinetic and pharmacodynamic changes due to volume overload, capillary leak, changes in protein-binding and interactions of multiple pharmacological agents [2]. In addition, extracorporeal procedures such as dialysis can lead to further alterations in pharmacokinetics and dynamics [3]. Antibiotic dosing during continuous venovenous dialysis is often challenging due to the need to account for dialysis parameters such as ultrafiltration, blood-, and dialysate flow [3]. The ADVanced Organ Support (ADVOS) multi system is an albumin-based hemodialysis device that can be used for liver and kidney support in critically ill patients, but also to correct acidosis and remove carbon dioxide (CO_2_) [2, 4, 5]. Besides having higher dialysis capacities than conventional continuous venovenous hemodialysis (CVVHD) (due to a larger surface area, higher blood-flow and dialysate flow limits), ADVOS combines conventional hemodialysis with albumin-based dialysis and acid-base regulation, potentially influencing both hydrophilic and protein-bound agents and adding another layer of complexity to the aforementioned dosing uncertainty [6]. Linezolid, a frequently used antibiotic in critically ill patients, is an oxazolidinone antibiotic that specifically targets gram positive bacteria including efficacy against methicillin-resistant *Staphylococcus aureus* (MRSA) and vancomycin-resistant enterococci (VRE). Due to its mainly non-renal elimination, linezolid is often utilized in patients with acute kidney injury and has a moderate plasma protein binding capacity of approximately 31% [7]. The minimum inhibitory concentration (MIC) of linezolid for gram-positive bacteria is variable in dependence of bacteria type [8]. For instance, MIC for Staphylococcus and Enterococcus species is described at 4 mg/L, while MIC for Streptococcus species is estimated at 2 mg/L according to the European committee on Antimicrobial Susceptibility Testing (EUCAST) [8, 9]. In general, trough serum concentrations (C_min_) between 2 and 7 mg/L and an area under the curve (AUC) over 24 h (AUC_24_) between 200 and 400 mg·h/L are recommended as an adequate target range [10, 11]. Inadequate drug exposure in critically ill patients could be associated with serious clinical consequences. Subtherapeutic concentrations may result in treatment failure and promote the emergence of antimicrobial resistance, while drug accumulation and overdosage can lead to adverse effects such as thrombocytopenia, anemia, rhabdomyolysis and interactions with other drugs [12, 13]. The probability of toxicity increases above C_min_ 7 mg/L, while C_min_ of ≥ 10 mg/L is considered toxic and associated with a high risk of hematological toxicity and hepatic injury [14].

In this prospective, observational study, we measured serum linezolid levels of critically ill patients, treated either with conventional CVVHD or ADVOS multi organ support and calculated total, extracorporeal and patient clearance in order to compare antibiotic treatment and elimination capacity between the two extracorporeal systems. To our knowledge, this is the first study to present in vivo data on linezolid pharmacokinetics in patients with albumin-based dialysis procedures.

## Materials and methods

### Study design

This prospective, monocentric observational study was performed at the Technical University of Munich (TUM) University Hospital, TUM School of Medicine and Health, a tertiary hospital in Munich, Germany. Patients were recruited between September 2024 and October 2025 at a single intensive care unit (ICU), specialized in gastroenterology and infectious diseases. The decision on the indication and form of the extracorporeal procedure was made at the discretion of the treating physicians independently of this study.

### Study population

All patients treated with linezolid and receiving extracorporeal treatment with either ADVOS or CVVHD were eligible for inclusion, provided written informed consent had been obtained from the patient or their legal representative.

### Interventions

Linezolid was dosed in adherence with general recommendation for patients on CVVHD, at 600 mg every 12 h and administered in a 300 mL solution. All patients received linezolid using an infusion pump, over a time period of 30 min via central venous catheter.

### Dialysis characteristics

Multifiltrate by Fresenius (Fresenius, Bad Homburg, Germany), equipped with an AV 1000 S Filter with 1.8 m^2^ of surface area, was used as CVVHD. ADVOS multi hemodialysis system (ADVITOS GmbH, Munich, Germany) was equipped with two ELISIO 19 H filters (Nipro D. Med Germany GmbH, Hamburg, Germany) for dialysis with a surface area of 1.9 m^2^ each and two ELISIO 11 H filters for albumin-recycling. Vascular access was predominantly femoral in both groups (Table 1).

**Table 1:** Patient baseline characteristics

Parameters	Total (*n* = 14)	ADVOS (*n* = 8)	CVVHD (*n* = 6)	*P* Value
Male, number (%)	8 (57.1%)	5 (62.5%)	3 (50%)	*p* = 0.64 (F)
Female, number (%)	6 (42.9%)	3 (37.5%)	3 (50%)	
Measured cycles, number	22	13	9	
Age (years), median [IQR]	55 [43–62]	51 [42–59]	59 [47–70]	*p* = 0.44 (MWU)
Weight (kg), median [IQR]	95 [71–100]	100 [74–104]	82 [71–98]	*p* = 0.43 (MWU)
Height (cm), median [IQR]	169 [165–179]	168 [165–177]	169 [167–178]	*p* = 0.64 (MWU)
BMI, median [IQR]	28.5 [25.7–36.3]	32.0 [25.7–36.7]	26.8 [25.3–33.2]	*p* = 0.48 (MWU)
Creatinine (mg/dL), median [IQR]	2.7 [1.8–3.7]	2.1 [1.5–3.1]	4.2 [2.4–6.7]	*p* = 0.09 (MWU)
Bilirubin (mg/dL), median [IQR]	5.8 [1.5–15.8]	6.7 [4.0–15.2]	3.5 [0.6–13.5]	*p* = 0.65 (MWU)
Albumin (g/dL), median [IQR]	3.2 [2.8–3.4]	3.2 [2.4–3.8]	2.9 [2.8–3.2]	*p* = 0.83 (MWU)
APACHE II Score, median [IQR]	11.5 [9.2–15.2]	12.5 [10.2–16.5]	10 [9.2–12.2]	*p* = 0.52 (MWU)
Reason for ICU admission, number (%) - ACLF - Sepsis - Pancreatitis	8 (57.1%) 4 (28.6%) 2 (14.3%)	5 (62.5%) 2 (25%) 1 (12.5%)	3 (50%) 2 (33.3%) 1 (16.7%)	
In-hospital mortality, number (%)	10 (71.4%)	6 (75%)	4 (66.7%)	*p* = 0.73 (F)
Blood flow (mL/min), mean ± SD		115 ± 37.6	100 ± 0	*p* = 0.13 (MWU)
Dialysate flow (mL/min), mean ± SD		197 ± 70.2	33.3 ± 0	*p* = 0.004 (MWU)
Ultrafiltration rate (mL/h), mean ± SD		131 ± 132	39 ± 33.3	*p* = 0.24 (MWU)
Vascular access, number (%)				
Femoral	11 (78.6%)	6 (75%)	5 (83.3%)	
Jugular	2 (14.3%)	2 (25%)	0	
Right atrial	1 (7.1%)	0	1 (16.7%)	

*CVVHD* continuous venovenous hemodialysis, *ADVOS* ADVanced Organ Support, *IQR* interquartile range, *SD* standard deviation, *BMI* body mass index, *ICU* intensive care unit, *ACLF* acute-on-chronic liver failure, *MWU* Mann-Whitney-U Test, *F* Fisher exact Test

### Sampling and drug measurements

Multiple measurements were performed during one measurement cycle, aiming for serum levels at 1, 2, 3, 4, 5, 7 and 11 h after antibiotic infusion. Because of operational challenges in intensive care practice, not all measurements could be performed consistently (supplemental Fig. 1). Multiple measurement cycles per patient were permitted and each was treated as an independent observation. Initial administrations of linezolid were not included in this analysis. Post-filter concentrations of linezolid were measured one hour after the linezolid infusion in order to calculate the machine clearance. Linezolid plasma concentration was determined by isotope dilution mass spectrometry (IDMS). The ClinMass^®^ TDM Kit System with Add-on Set Antibiotics was used for the quantitative determination of linezolid in serum by liquid chromatography tandem mass spectrometry (LC-MS/MS) (RECIPE Chemicals + Instruments GmbH München/Germany). The kit is CE-IVD-certified, REF MS9000 and MS9700 were used.

Blood was drawn into serum vials via arterial access in tubes without gel separators and centrifuged immediately (10 min at 3,000 × g). Some gels can partially adsorb the analytes, leading to falsely low analysis values. Sample preparation was carried out by protein precipitation adding Internal Standards (IS). Briefly, 50 µL of serum sample and 100 µL IS, reconstituted with precipitant P, were mixed vigorously for 30 s on a vortex mixer. The samples were centrifuged at 10,000 x g for five minutes. 50 µL of the supernatant was transferred into autosampler vials and diluted with 950 µL LC-MS grade H2O. 10 µL were injected into the mass spectrometry system. An AB Sciex API 5500 Qtrap LC/MS/MS (AB Sciex LLC, Framingham, MA, USA) equipped with an Agilent 1200 HPLC system (Agilent, Santa Clara, CA, USA) was used in multiple reaction mode (MRM) as mass spectrometry system to determine linezolid plasma concentration. A full calibration with four calibration levels was carried out within each run. Two control samples with different levels were also included. Data were evaluated by Analyst 1.7.2. using the matching IS.

### Clearance calculations

Total systemic clearance (CL_total_) was calculated based on the AUC of serum concentrations (C) over time (t). Only measurement cycles with at least five chronologically plausible values were applied for AUC calculations. Measurements were excluded if serum-concentration reached a second peak during the cycle, indicating off-scheduled or belated antibiotic infusions.

1 $$\:{CL}_{total}\:=\frac{\:dose\:}{AUC}$$

AUC was estimated using the trapezoidal rule (Eq. 2):

2 $$\:AUC\:\approx\:\sum_{}\:\:\left[\frac{\left(C_i\:+\:C_{i+1}\right)}2\right]\:\times\:(t_{i+1}\:-\:t_i)$$

For machine clearance (CL_mach_), diffusive and adsorption clearance were calculated. The convective contribution to solute clearance was negligible compared to the diffusive clearance of CVVHD since ultrafiltration rates were low and only used for fluid balancing. Convection was therefore not taken into account when calculating clearance. Machine clearance was calculated using blood flow (Q_blood_) and simultaneously measured pre- (C_pre_) and post-filter (C_post_) concentrations (Eq. 3).

3 $$\:C{L}_{mach}={Q}_{blood}\times\:\:\:\frac{\left({C}_{pre}-\:{C}_{post}\right)}{{C}_{pre}}$$

Patient clearance (CL_patient_) was calculated by subtracting machine clearance (CL_mach_) from total clearance (CL_total_) (Eq. 4).

4 $$\:{CL}_{patient}\:=\:{CL}_{total}\:-\:{CL}_{mach}$$

### Trough levels and AUC_24_

Trough serum concentrations (C_min_) were measured at hour 11 of antibiotic therapy. C_min_ below 2 mg/L were considered definitely below MIC for gram-positive bacteria [8, 9, 14], and C_min_ above 10 mg/L were considered toxic, with a high risk of hematological toxicity and hepatic injury [14, 15]. Drug exposure over a 12-hour dosing interval was estimated by calculating AUC_12_ using the trapezoidal rule. Missing concentrations at hour 12 were extrapolated using log-linear terminal extrapolation. Reflecting the repeating dosing under steady-state assumptions, concentrations at hour 0 were set equal to the extrapolated concentration at hour 12. There were no changes in linezolid-dosage. AUC_24_ was estimated by doubling calculated AUC_12_. Extrapolated data was only employed in estimation of AUC_24_ – not in calculations of clearance and trough levels.

Monte-Carlo simulation-based pharmacokinetic/pharmacodynamic analysis, underlying EUCAST breakpoint considerations suggests a target attainment probability of 83% for staphylococci at an AUC_24_/MIC target of 100, assuming a MIC of 2 mg/L [9]. In clinical studies, these target ranges have been shown to reach microbiological eradication rates of 80% in sepsis patients, supporting the set threshold [16]. Consequently, AUC_24_ estimates below 200 mg·h/L were considered as subtherapeutic, estimates between 200 and 400 mg·h/L within target range, and estimates above 400 mg·h/L associated with higher toxicity [9–11].

### Statistical analysis

All statistical analyses were performed using R version 4.4.2 (R Foundation for Statistical Computing, Vienna, Austria). Continuous variables were summarized as medians with interquartile ranges, while categorical variables were presented as absolutes and relative frequencies. Due to a non-normal distribution and outliers in clearance parameters, comparisons between the dialysis modalities were conducted using Wilcoxon rank-sum test. Frequencies were compared using the Fisher’s exact test. All tests were two-sided, and a p-value below 0.05 was considered statistically significant. First-order elimination was assessed by linear regression of log-transformed elimination-phase concentrations (from 2 h, as the peak occurred at 1 h) against time, deriving R² and the elimination half-life per cycle. The association between C_min_ and the AUC_1–11_ from measured concentrations was evaluated by Spearman correlation. In an exploratory analysis, concentrations were normalized to relative body mass (measured concentration × individual/mean cohort weight), and the coefficient of variation at each sampling time was compared before and after normalization using the Wilcoxon signed-rank test. To address repeated measurements, a sensitivity analysis restricted to one cycle per patient was performed.

## Results

Data were collected over a period of 14 months, from September 2024 to October 2025. A total of 29 measurement cycles was performed. After excluding invalid measurement cycles, most often due to inadequate increases of concentration levels, 22 measurement cycles were included in the analysis. Of the 176 scheduled measurements among the 22 included cycles (8 per cycle), 147 (83.5%) were successfully obtained and included in the study. Distribution of missing measurements is described in supplemental Fig. 1.

### Patient characteristics

Baseline characteristics are presented in Table 1. Measurements were performed in 14 patients (6 female). Eight received ADVOS-dialysis, while six received CVVHD. Median age was 55 [43–62] years. Both baseline bilirubin and APACHE II Score were similar between extracorporeal procedures. Baseline creatinine was higher in CVVHD, though not significantly. Acute on chronic liver failure (ACLF) was the most common reason for ICU-treatment at 57.1%, followed by sepsis at 28.6% and pancreatitis at 14.3%. A mean blood flow of 115 mL/min (SD 37.6) and dialysate flow of 197 mL/min (SD 70.2) was utilized in ADVOS, while CVVHD was employed steadily at a blood flow of 100 mL/min and a dialysate flow of 33.3 mL/min. Mean ultrafiltration rate was 131 mL/h (SD 132) for ADVOS and 39 mL/h (SD 33.3) for CVVHD.

### Pharmacokinetic characteristics

Elimination-phase concentrations declined log-linearly in all cycles (median R² = 0.98, IQR 0.96–0.99; R² > 0.90 in 95% of cycles), with a median elimination half-life of 6.6 h (IQR 5.1–7.6), supporting first-order elimination kinetics (Supplemental Fig. 3). A median of 13 doses [IQR 6–17] had been administered before the analyzed dose, corresponding to a median of 156 h of therapy and approximately 24 elimination half-lives. As at least three prior doses had been given in every cycle (range 3–22), corresponding to more than five half-lives, beyond the four to five conventionally required – steady-state conditions could be assumed in all cycles. Weight normalization reduced the mean coefficient of variation across sampling time points from 0.51 to 0.46 (− 11%, Wilcoxon signed-rank *p* = 0.016), indicating a significant but modest contribution of body weight to the observed variability (Supplemental Fig. 2, Supplemental Table 1).

### Comparison of clearance

**Fig. 1:**
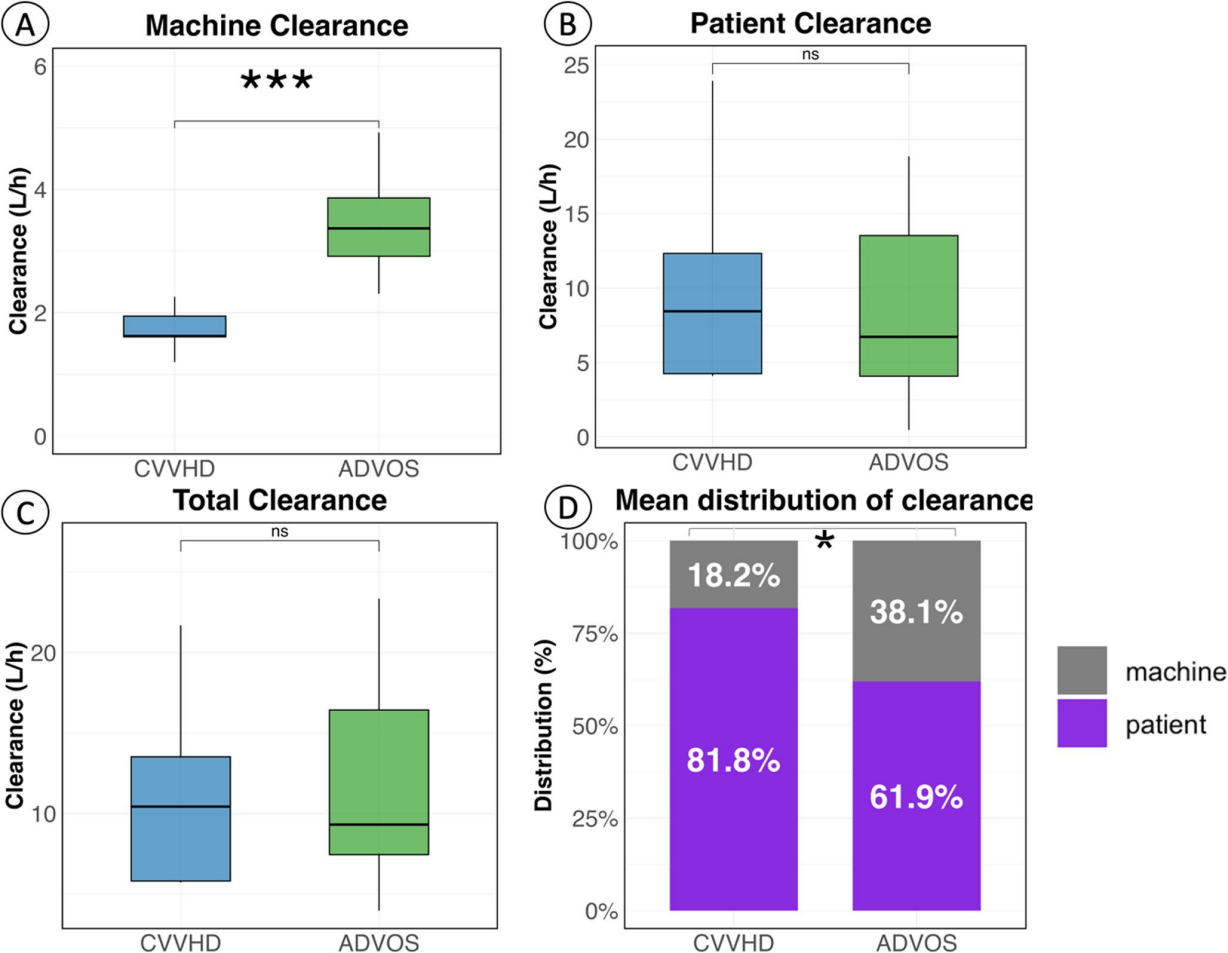
Machine Clearance (**A**), Patient Clearance (**B**), Total Clearance (**C**) and mean distribution of clearance (**D**), compared between CVVHD and ADVOS. Boxplots in **A**–**C **show absolute clearance values; the stacked bars in **D **show mean proportional shares, which boxplots cannot adequately represent. * indicating statistical significance (*p* < 0.05); ***indicating statistical significance (*p* < 0.001), CVVHD: continuous venovenous hemodialysis, ADVOS: ADVanced Organ Support

Estimated total clearance by AUC-method was not significantly different in patients who received ADVOS-Dialysis compared to CVVHD (*p* = 0.95, Wilcoxon test) (see Table 2). There was also no difference in estimated patient clearance between patients treated with CVVHD compared to those being treated by ADVOS dialysis (*p* = 0.69, Wilcoxon test) (Table 2). Machine clearance, however, showed a significantly higher median clearance rate in ADVOS dialysis at 3.4 [2.9–3.9] L/h compared to CVVHD at 1.6 [1.6–1.9] L/h (*p* < 0.001, Wilcoxon test) (Table 2). In a sensitivity analysis restricted to one cycle per patient (*n* = 14), machine clearance remained significantly higher in ADVOS than in CVVHD (median 3.24 vs. 1.78 L/h, *p* = 0.0007), confirming that this difference was not driven by repeated measurements. When calculating the proportion of machine and patient clearance to total clearance for each patient, machine clearance accounted for 38.1% of total clearance in ADVOS, which was significantly higher compared to 18.2% in CVVHD (Fig. 1) (*p* = 0.038, Wilcoxon rank sum test). The trough concentration correlated strongly with the AUC over the measured sampling interval (AUC₁₋₁₁; Spearman rho = 0.93); as the trough is the final measured point contributing to this AUC, the association is partly structural, but the residual variability indicates that Cmin provides a useful practical estimate of overall exposure.

**Table 2:** Median and mean clearance, compared between CVVHD and ADVOS

Clearance	Dialysis	Median clearance	IQR	Mean clearance	SD	*p*-value
Total (L/h)	CVVHD	10.4	5.8–13.5	12.1	7.3	0.95
	ADVOS	9.3	7.4–16.3	12.0	6.3	
Patient (L/h)	CVVHD	8.4	4.3–12.3	10.4	7.2	0.69
	ADVOS	6.7	4.1–13.5	8.6	6.1	
Machine (L/h)	CVVHD	1.6	1.6–1.9	1.7	0.3	< 0.001
	ADVOS	3.4	2.9–3.9	3.4	0.8	

*CVVHD* continuous venovenous hemodialysis,* ADVOS* ADVanced Organ Support, *IQR* interquartile range, *SD* standard deviation

### Linezolid trough levels (C_min_) and estimated AUC_24_ in regards to target range

**Fig. 2:**
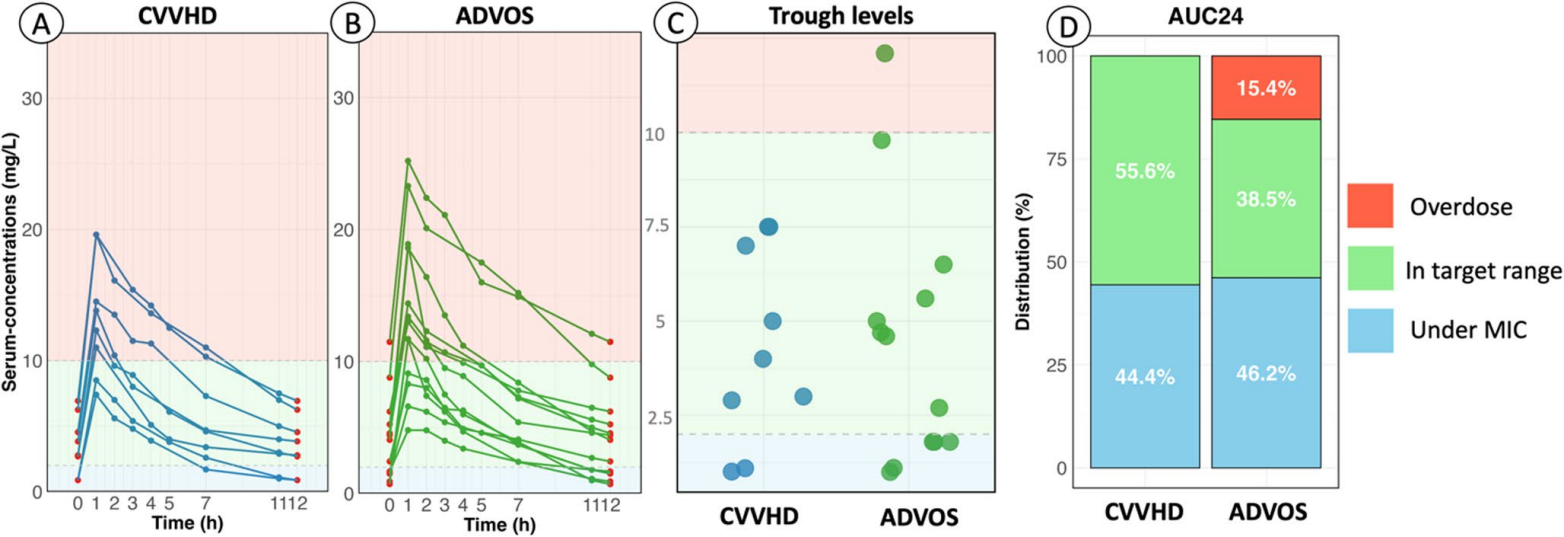
Linezolid serum concentrations in relation to measurements over time between CVVHD (**A**) and ADVOS (**B**), extrapolated data highlighted in red, (**C**) Trough levels and (**D**) AUC24 in relation to target range. Abbreviations: CVVHD: continuous venovenous hemodialysis, ADVOS: ADVanced Organ Support, AUC: area under the curve, MIC: minimum inhibitory concentrations

Cmin was measured 11 h after linezolid administration. In CVVHD, 2 out of 9 (22.2%) measurements were below MIC. In ADVOS, 5 out of 13 (38.5%) measurements were below MIC and 1 out of 13 (7.7%) measurements fell within the toxic range (Fig. 2; Table 3). The median estimated AUC24 did not differ significantly between CVVHD and ADVOS (Table 3). In ADVOS, among the 13 estimated AUC24, 6 (46.2%) were below, 5 (38.5%) within, and 2 (15.4%) above the target range (Fig. 2; Table 3). In CVVHD, 4 of 9 (44.4%) estimated AUC24 values were below and 5 of 9 (55.6%) within the intended range (Fig. 2; Table 3). Statistical comparisons of trough levels and AUC24 between CVVHD and ADVOS were not significantly different. AUC24 below the target range did not correlate significantly with mortality (Fisher’s exact, OR = 1.93, *p* = 0.65).

**Table 3:** Comparison of trough level and comparison of estimated AUC_24_ between dialysis type

	Dialysis	Median	IQR	p-value	Measurements in relation to target range
Trough level (mg/L)	CVVHD & ADVOS	4.3	1.8–6.3		> 10 mg/L: 1/22 (4.5%) 2–10 mg/L: 14/22 (63.6%) < 2 mg/L: 7/22 (31.8%)
	CVVHD	4.0	2.9–7.0	0.39	> 10 mg/L: 0/9 (0%) 2–10 mg/L: 7/9 (77.8%) < 2 mg/L: 2/9 (22.2%)
	ADVOS	4.6	1.8–6.2		> 10 mg/L: 1/13 (7.7%) 2–10 mg/L: 7/13 (53.8%) < 2 mg/L: 5/13 (38.5%)
Estimated AUC_24_ (mg·h/L)	CVVHD & ADVOS	236	111–324		> 400 mg·h/L: 2/22 (9.1%) 200–400 mg·h/L: 10/22 (45.5%) < 200 mg·h/L: 10/22 (45.5%)
	CVVHD	221.2	165.6–374.9	0.94	> 400 mg·h/L: 0/9 (0%) 200–400 mg·h/L: 5/9 (55.6%) < 200 mg·h/L: 4/9 (44.4%)
	ADVOS	250.9	110.0–299.4		> 400 mg·h/L: 2/13 (15.4%) 200–400 mg·h/L: 5/13 (38.5%) < 200 mg·h/L: 6/13 (46.2%)

*CVVHD* continuous venovenous hemodialysis, *ADVOS* ADVanced Organ Support, *IQR* interquartile range, *AUC* area under the curve

## Discussion

In this study it was shown that the albumin based ADVOS dialysis system has a significantly higher in vivo machine clearance of linezolid compared to CVVHD. Similar results have been described in vitro by König C et al. [6] with a clearance rate between 2.79 L/h and 4.65 L/h in ADVOS depending on blood flow levels. Due to its small molecular mass (337.35 g/mol) and predominantly protein unbound state in serum (70%), machine clearance of linezolid is highly affected by size of dialyzer surface, dialysate flow and blood flow levels [7]. In counter-current dialysis, the diffusive clearance of a small, freely diffusible solute cannot exceed the lower of blood flow and dialysate flow (Michaels relationship). In CVVHD, the dialysate flow of 33.3 mL/min (2.0 L/h) constituted the limiting factor, and the measured machine clearance (1.6–1.9 L/h) was already close to this ceiling; in ADVOS, the approximately six-fold higher dialysate flow (197 vs. 33.3 mL/min), rather than the only marginally higher blood flow (115 vs. 100 mL/min), accounts for the higher machine clearance [17, 18]. The markedly higher dialysate flow required by ADVOS, while improving extracorporeal clearance, also causes greater water and energy consumption. This trade-off is of growing relevance in light of current efforts towards more sustainable, low-flow dialysis [19, 20], and should be weighed against the clearance benefit when selecting an extracorporeal modality. In addition to a higher capacity to dialyze water soluble substances, ADVOS also targets linezolid in the albumin-bound state, causing an even higher clearance rate [6]. While estimated total clearance did not differ between dialysis methods in this study, machine clearance accounted for a significantly greater share of total clearance in patients receiving ADVOS treatment. This dissociation likely reflects the large interindividual variability in patient clearance, the limited sample size, and the fact that machine clearance represented only a minority of total clearance (18.2% in CVVHD and 38.1% in ADVOS), so that differences in the extracorporeal component were diluted at the level of total clearance. In addition, patient clearance tended to be numerically lower in ADVOS (median 6.7 vs. 8.4 L/h), suggesting a possible compensatory contribution, though not statistically significant. In order to counteract the higher machine clearance, ADVITOS-GmbH suggests an increase in dosage from 600 mg linezolid every 12 h to 600 mg linezolid every 8 h. Examining the estimation of AUC_24_ in all patients, increasing the dosage frequency may be beneficial for both dialysis methods, in avoiding subtherapeutic serum levels, which were observed in a substantial proportion of measurements in both groups. However, this approach may also increase the risk of toxic serum levels, which were also observed in ADVOS, indicating high interindividual variability. Part of this variability is expected from the range of body size after a fixed 600 mg dose; weight normalization reduced the variability significantly but only modestly, indicating that body size is a minor determinant of exposure and reinforcing the need for therapeutic drug monitoring rather than size-based dosing alone. As an alternative approach, Wicha et al. [21] proposed the administration of linezolid via continuous infusion, a method that has been demonstrated to reduce the likelihood of Grade 3 thrombocytopenia. Similar to the presented study, Wicha et al. and Zoller et al. [14, 21] observed a high interpersonal variability of linezolid pharmacokinetics, with a substantial amount of both subtherapeutic and toxic levels of linezolid especially in patients with continuous renal replacement therapy, extracorporeal lung assistance and lung- or liver-transplanted patients. According to the authors, general therapeutic drug monitoring is suggested in critically ill patients being treated with linezolid in order to improve outcome by preventing subtherapeutic serum-levels, limiting the development of antimicrobial resistance and avoiding complications due to toxicity [22]. This study underscores the necessity of therapeutic drug monitoring (TDM) in critically ill patients with extracorporeal procedures, by observing a substantial amount of inadequately treated patients (45.5% by AUC_24_ and 31.8% by C_min_) in both CVVHD and ADVOS.

Our study has several limitations. First, due to the infrequent application of ADVOS and consequently limited sample size, this study may be underpowered to detect clinically significant differences. Nevertheless, it provides novel in-vivo data on linezolid-pharmacokinetics in albumin-dialysis. Secondly, although all measurement cycles were treated separately, some were taken from the same patient in order to maximize serum measurements in a limited cohort—which may distort the final results. A sensitivity analysis restricted to one cycle per patient confirmed, however, that the higher machine clearance in ADVOS persisted. The choice of modality was furthermore made at the discretion of the treating physicians rather than by randomization, which may introduce selection bias, although baseline characteristics were comparable between groups (Table 1). Furthermore, 7 of 29 total measurement cycles had to be excluded from the study due to invalid results – representing the difficulties of an observational study taking place in parallel to patient treatment, with postponed or advanced antibiotic treatment due to outside factors. Furthermore, a wide variability of AUC led to a noticeable scattering of calculated total and patient clearances, potentially masking effects of dialysis-method on total clearance. This observation is similar to Zoller et al. [14], and marks the wide interpersonal difference in ICU-patients. The employment of a creatinine-based estimation, similar to Wicha et al. and Totschnig et al. [21, 23], was discussed but finally decided against in order to depict real-world data. Moreover, AUC_24_ was not measured but estimated by doubling AUC_12_, which was calculated from measured serum concentrations and extrapolated data. Machine clearance was further derived from pre- and post-filter concentrations measured at a single time point and assumed constant over the interval; temporal changes in filter performance and adsorption cannot be excluded. As linezolid distributes into total body water with only moderate protein binding, incomplete equilibration across the erythrocyte membrane during filter transit could also have influenced the blood-side clearance estimate, and dialysate-side measurement would be preferable in future studies. Finally, no pre-dose trough was measured, so the volume of distribution could not be derived; although the number of preceding doses supported steady-state conditions, this was not verified by a measured pre-dose concentration.

In conclusion, this study describes a significantly higher machine clearance in patients being treated with ADVOS compared to conventional CVVHD, while patient clearance and total clearance were similar in both dialysis types. Despite the higher machine clearance in ADVOS, C_min_ and estimated AUC_24_ did not differ significantly. A substantial amount of measurement cycles was outside of target range in both ADVOS and CVVHD, emphasizing the necessity of TDM in ICU-patients being treated with extracorporeal procedures. Future studies are needed to compare alternative dosage regimens, such as more frequent, continuous or prolonged application of linezolid under TDM between ADVOS and CVVHD [21].

## Supplementary Information

Below is the link to the electronic supplementary material.

Supplementary Material 1

## Data Availability

The datasets used and/or analysed during the current study are available from the corresponding author on reasonable request.

## Glossary

ACLF: acute-on-chronic liver failure
AUC: area under the curve
AUC_1-11_: area under the curve over the measured sampling interval (1–11 h)
AUC_12_: area under the curve over 12 hours
AUC_24_: area under the curve over 24 hours
BMI: body mass index
C: serum concentrations
C_min_: trough concentration
CL_mach_: machine clearance
CL_patient_: patient clearance
CL_total_: total systemic clearance
CO_2_: carbon dioxide
C_pre_: pre-filter concentrations
C_post_: post-filter concentrations
CVVHD: continuous venovenous hemodialysis
EUCAST: European committee on Antimicrobial Susceptibility Testing
ICU: intensive care unit
IQR: interquartile range
MIC: minimum inhibitory concentrations
Q_blood_: blood flow
SD: standard deviation
t: time
TDM: therapeutic drug monitoring
