# Supplementary material for: Serum concentrations and pharmacokinetics of linezolid in critically ill patients dialyzed by ADVanced Organ Support compared to conventional continuous renal replacement therapy
Source: Eur J Clin Pharmacol. 2026 Jul 10;82(8):199. doi: 10.1007/s00228-026-04129-0 (PMC13350108; doi:10.1007/s00228-026-04129-0)
Supplement: Supplementary file 1 — Supplementary Material 1 [file 228_2026_4129_MOESM1_ESM.docx]

**Serum concentrations and pharmacokinetics of linezolid in critically ill patients dialyzed by ADVanced Organ Support compared to conventional continuous renal replacement therapy**

Julian Triebelhorn^1^, Johanna Erber^1^, Heike Schneider^2^, Jochen Schneider ^1^, Laura Wagner^1^, Yizhu Li^1^, Eva Ortner^1^, Roland M. Schmid^1^, Ulrich Mayr^1^, Tobias Lahmer^1^, Miriam Dibos^1*^

^1^ TUM School of Medicine and Health - Clinical Department of Internal Medicine II, TUM University Hospital, Munich, Germany

^2^ TUM School of Medicine and Health - Clinical Department of Clinical Chemistry and Laboratory Medicine, TUM University Hospital, Munich, Germany

* Corresponding author

**Corresponding author:**

Miriam Dibos

TUM School of Medicine and Health - Clinical Department of Internal Medicine II

TUM University Hospital

Ismaninger Str. 22

81675 Munich

Tel: +49 89 4140 5847

**Supplemental material**

Supplemental Figure 1: Missing data by measurements and dialysis type


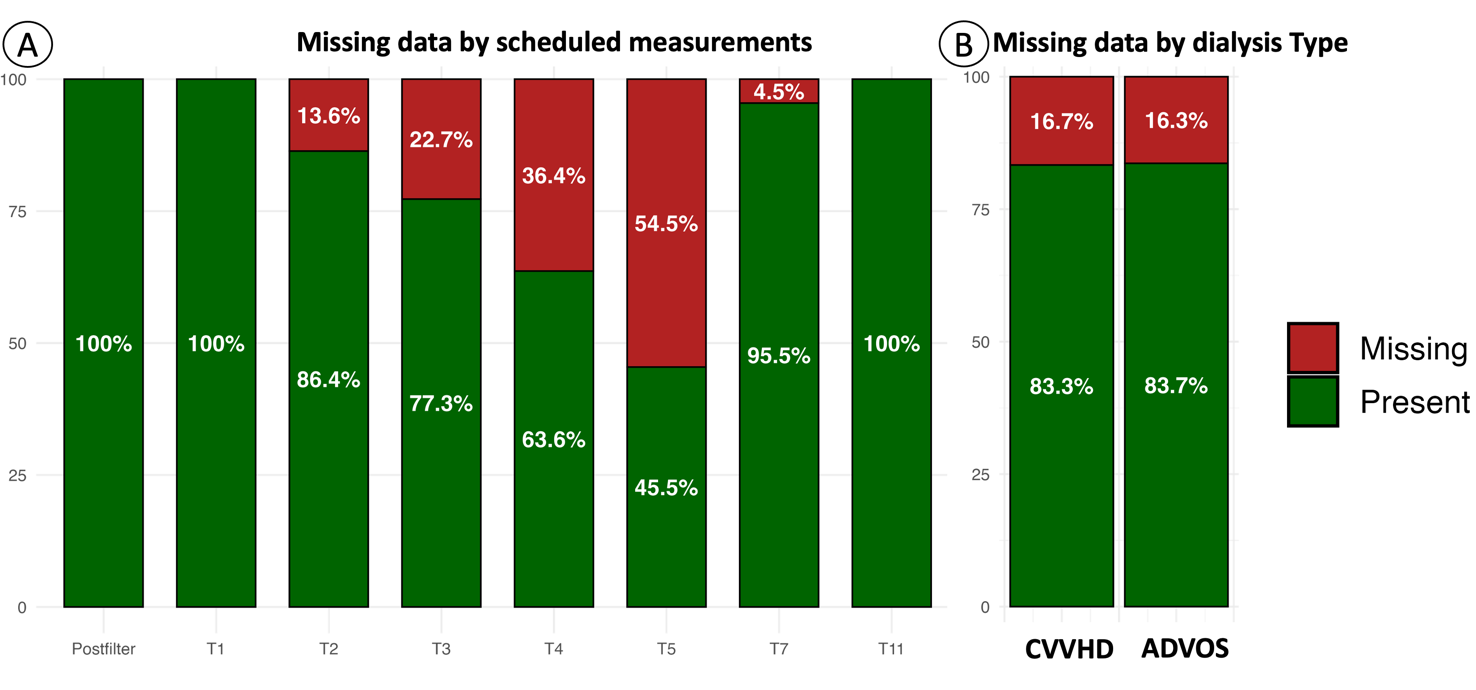


***Supplemental Figure 2: Coefficient of variation (CV) of linezolid serum concentrations at each sampling time before (raw) and after weight normalization, showing a significant but modest reduction (mean CV 0.513 → 0.457, −11.0 %; Wilcoxon signed-rank p = 0.016).***

***
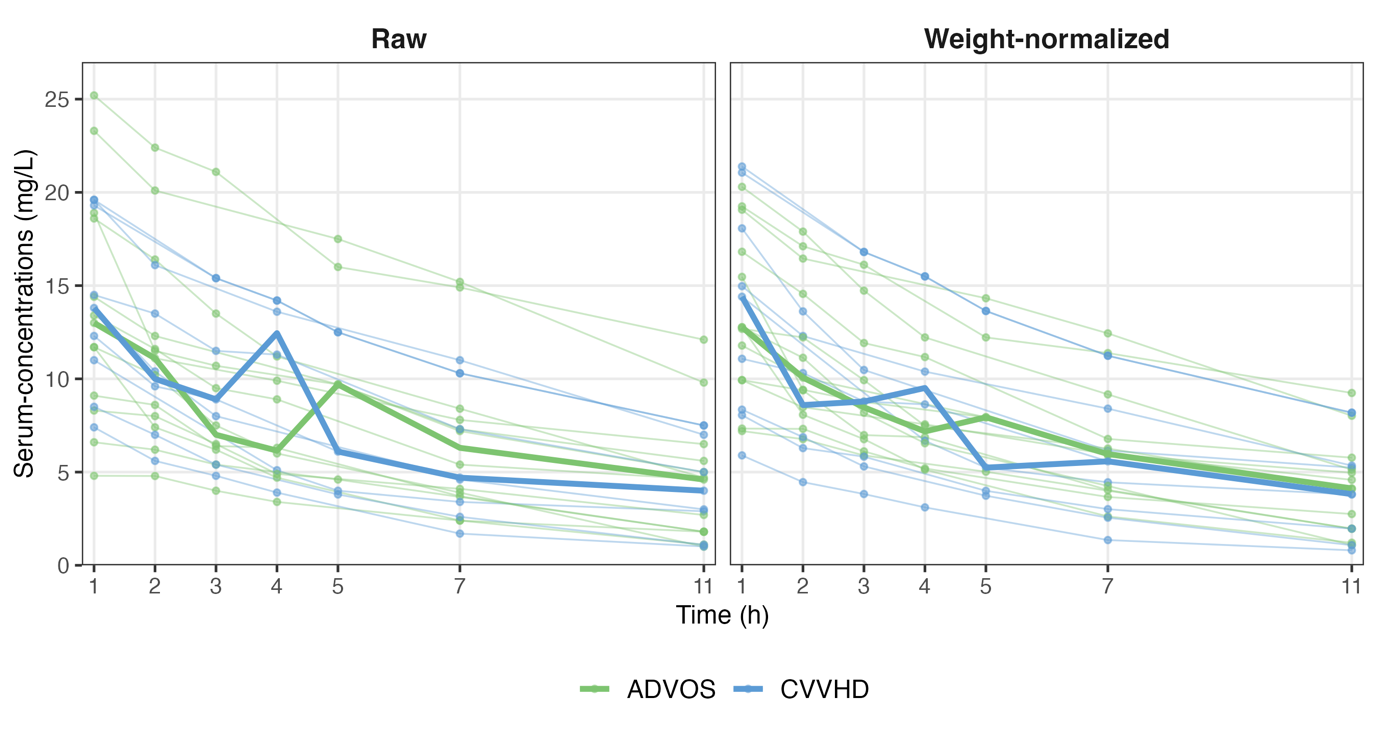
***

***Supplemental Figure 3: Semi-logarithmic plot of linezolid elimination-phase concentrations (from 2 h after infusion) over time for all 22 measurement cycles, confirming first-order elimination (median R² = 0.98, IQR 0.96 – 0.99; median half-life 6.6 h)***

*
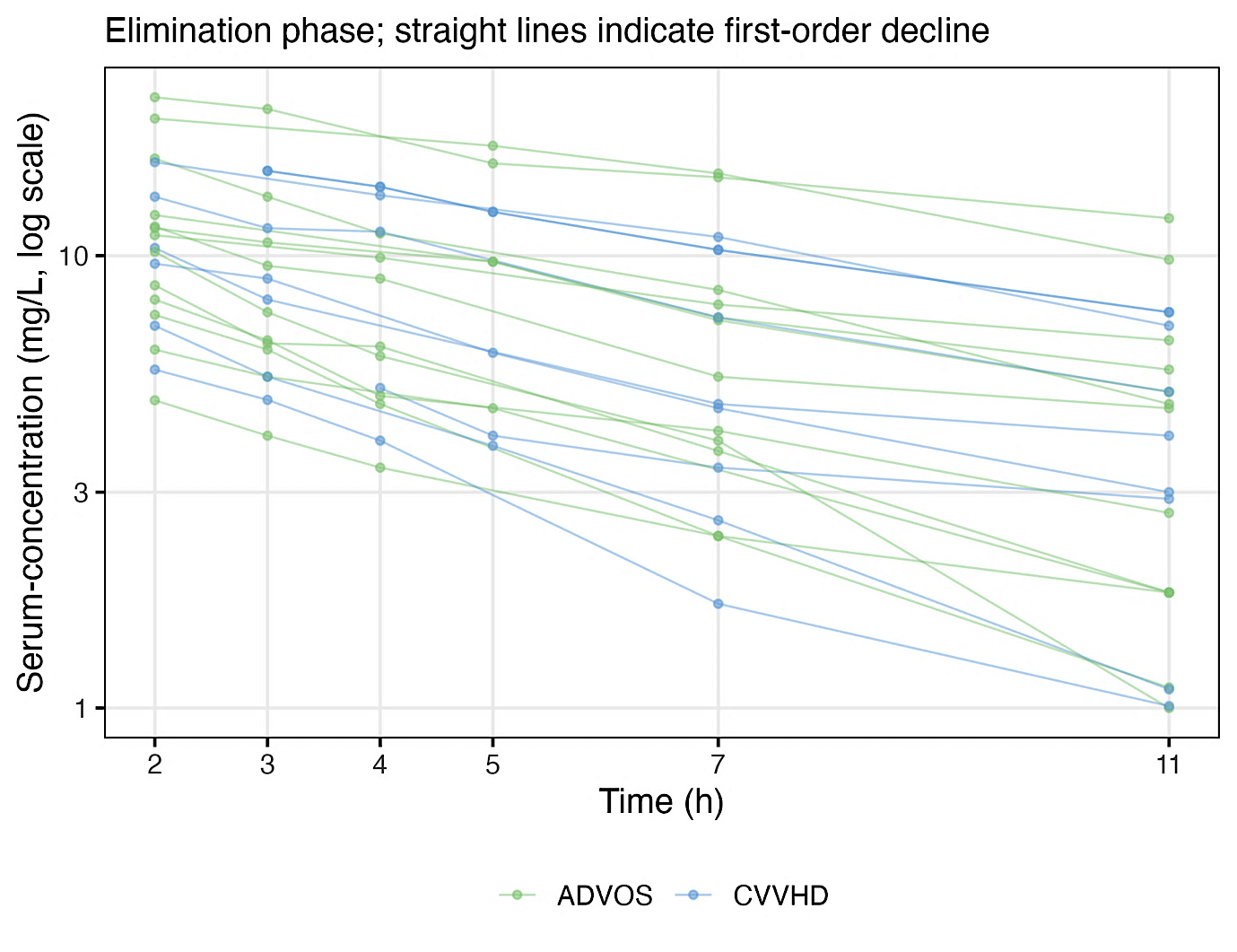
*

***Supplemental Figure 4: Correlation between trough concentration (Cmin) and the area under the curve over the measured sampling interval (AUC1–11) across all 22 measurement cycles, by dialysis modality (ADVOS green, CVVHD blue). Spearman rho = 0.93 overall, with no significant difference between modalities (Fisher r-to-z, p = 0.23)****
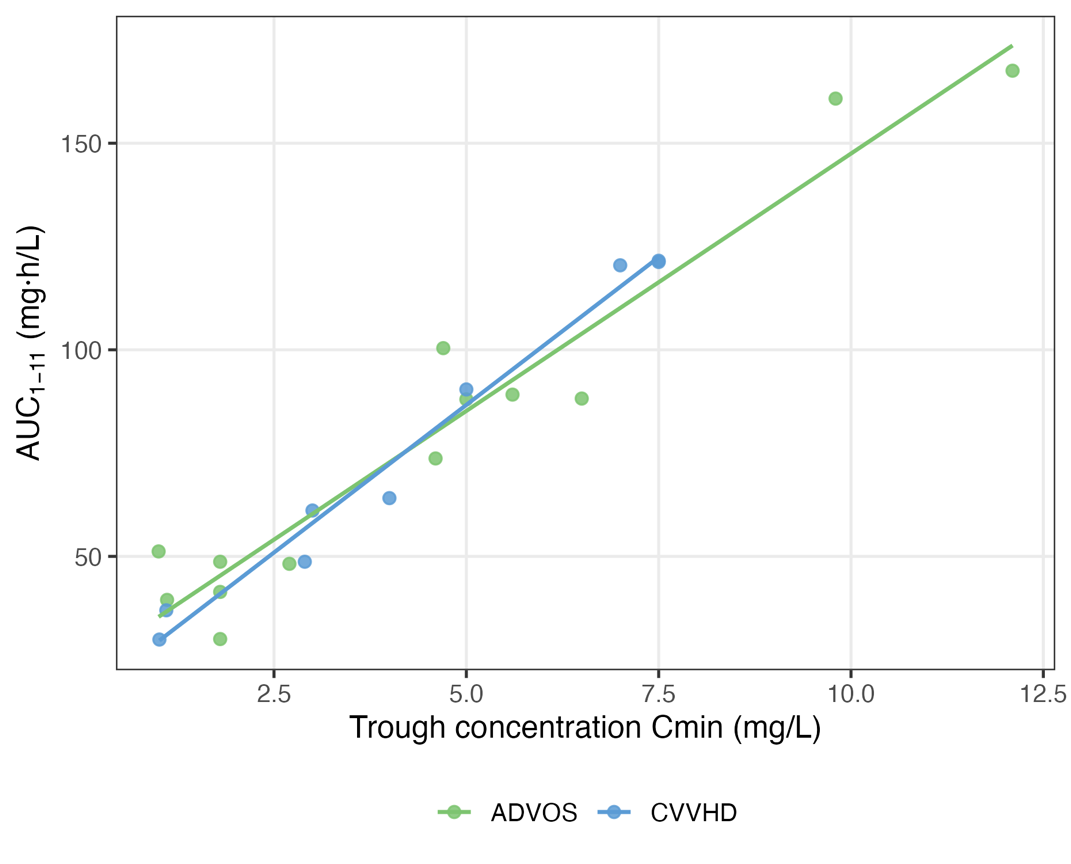
*

***Supplemental Table 1: Coefficient of variation before and after weight normalization:*** Coefficient of variation (CV) of linezolid serum concentrations at each sampling time, before and after weight normalization. Mean CV decreased from 0.513 to 0.457 (-11.0%); Wilcoxon signed-rank p = 0.016.

| Time (h) | n | Mean raw (mg/L) | CV raw | Mean norm. (mg/L) | CV normalized | CV change (%) |
| --- | --- | --- | --- | --- | --- | --- |
| 1 | 22 | 13.86 | 0.40 | 13.57 | 0.36 | -10.2 |
| 2 | 19 | 11.2 | 0.43 | 10.67 | 0.36 | -15.1 |
| 3 | 17 | 9.42 | 0.50 | 9.6 | 0.44 | -10.5 |
| 4 | 14 | 8.4 | 0.47 | 8.71 | 0.43 | -8.5 |
| 5 | 10 | 9.64 | 0.52 | 8.77 | 0.50 | -4.9 |
| 7 | 21 | 6.6 | 0.60 | 6.31 | 0.51 | -14.4 |
| 11 | 22 | 4.43 | 0.69 | 4.24 | 0.6 | -12.5 |
| Mean (all) | **125** | **–** | **0.51** | **–** | **0.46** | **-11** |
